# A Randomized Controlled Trial of the Safety and Efficacy of Systemic Enzyme Supplementation on Symptoms and Quality of Life in Patients with Idiopathic Pulmonary Fibrosis

**DOI:** 10.3390/diseases12070155

**Published:** 2024-07-13

**Authors:** Neha Shah

**Affiliations:** Pulmonary Fibrosis NOW, Chino, CA 91710, USA; drnehashah772@gmail.com

**Keywords:** idiopathic pulmonary fibrosis, quality of life, complementary treatment, systemic enzyme supplements

## Abstract

Idiopathic pulmonary fibrosis (IPF) imposes a substantial symptom burden that adversely impacts patients’ quality of life. Current anti-fibrotic treatments for IPF provide limited symptomatic relief, necessitating the implementation of complementary disease management strategies to enhance health-related quality of life (HRQOL). Serracor-NK^®^ and Serra Rx260, systemic enzyme supplements, improved symptoms and HRQOL with favorable safety profiles in a proof-of-concept study in PF patients. This prospective, double-blind randomized placebo-controlled trial enrolled 100 IPF patients from six institutions. The supplement group (*n* = 50) received the oral systemic enzyme supplements Serracor-NK^®^ and Serra Rx260 in addition to standard care for 6 months, while the placebo group (*n* = 50) received standard care alone. The primary objective was to evaluate the regimen’s impact on symptoms, QOL, and well-being using the UCSD shortness of breath (UCSD-SOB) questionnaire, St. George’s respiratory questionnaire (SGRQ), and WHO well-being index (WHO-5). Safety evaluation was a secondary objective. A significantly higher proportion of patients in the supplement group demonstrated meaningful improvement in symptoms as compared to the placebo group, as assessed by the UCSD-SOB (*p* < 0.05) and SGRQ questionnaires (*p* < 0.05). Additionally, a significantly greater proportion of patients in the supplement group showed improved QOL and well-being (*p* < 0.05) and reduced health impairment (*p* < 0.05), as assessed by SGRQ. Mental well-being (WHO-5) and physical activity (SGRQ activity domain) did not differ significantly between the groups. Safety assessments, including liver function tests and vital signs, indicated that the supplement regimen was well tolerated. To conclude, Serracor-NK^®^ and Serra Rx260 alleviate symptoms and enhance HRQOL in IPF patients with a favorable safety profile (Clinical Trials Registry India registration number: CTRI/2020/05/025374).

## 1. Introuction

Idiopathic pulmonary fibrosis (IPF) is a progressive interstitial lung disease characterized by chronic and irreversible scarring of the lung tissue. It imposes a significant symptom burden on patients, leading to a marked decline in health-related quality of life (HRQOL). Patients with IPF commonly experience a range of debilitating symptoms, including chronic exertional dyspnea, persistent dry cough, and overwhelming fatigue. Dyspnea, a hallmark manifestation of the disease, is not only prevalent [1] but also extremely distressing for patients. The chronic cough associated with IPF can severely disrupt daily life by interfering with sleep, speech, and social activities while also contributing to complications such as desaturation, musculoskeletal pain, and urinary incontinence. Furthermore, the psychological impact of IPF should not be overlooked, as patients frequently experience feelings of fear, anxiety, hopelessness, and helplessness [2]. Given the multifaceted challenges faced by patients with IPF, it is imperative to focus on comprehensive symptom assessment and management strategies to improve patient outcomes.

Improving symptoms and QOL domains like physical functioning, social participation, and emotional well-being, beyond mere survival, remains a critical objective in the management of IPF [3]. Patient-reported outcomes (PROs) play a crucial role by providing direct insights into patients’ perceptions of their health status and QOL in the context of their disease and its treatments [4]. Various specific and generic instruments have been validated for assessing symptom burden and HRQOL in IPF patients, offering valuable tools to quantify health improvements resulting from therapeutic interventions.

The current standard therapies for IPF are the oral antifibrotic drugs pirfenidone and nintedanib, which aim to slow disease progression by reducing the rate of lung function decline but offer limited improvement in HRQOL. These treatments are associated with adverse effects including nausea, diarrhea, loss of appetite, lethargy, and photosensitivity, which further compromise QOL and often lead to treatment discontinuation. The long-term adherence to these drugs is variable [5], and suspension of treatment due to the occurrence of adverse reactions leads to significant functional decline [6]. Therefore, exploring supplementary or complementary therapies that enhance HRQOL in addition to improving clinical outcomes is crucial.

Serrapeptase and nattokinase are systemic enzymes known for their fibinolytic, anti-inflammatory, and immunomodulatory properties and have demonstrated efficacy in the breakdown of fibrous scar tissue [7,8]. These enzymes present a promising adjunctive approach to the management of IPF. In our previous proof-of-concept study, a 12-week regimen of the oral systemic enzyme supplements Serracor-NK^®^ and Serra Rx260 showed significant improvement in symptoms, mental and physical well-being, and HRQOL in patients diagnosed with either PF (31%) or IPF (69%) [9]. The current randomized controlled trial was designed to further validate the safety and efficacy of Serracor-NK^®^ and Serra Rx260 in improving symptoms and HRQOL, specifically in patients with IPF.

## 2. Materials and Methods

### 2.1. Study Design and Oversight

This was a prospective, interventional, double-blind, randomized, placebo-controlled parallel group clinical study performed at six institutions in India (Clinical Trials Registry India, registration number: CTRI/2020/05/025374). The study was conducted in accordance with the ethical principles outlined in the current revision of the “Declaration of Helsinki 2013” and adhered to the guidelines set forth by the Indian Council of Medical Research (ICMR) for biomedical research on human subjects, new drugs and clinical trials rules 2019 of Indian Pharmacopoeia and Cosmetics Act, ICH-GCP guidelines, and following all applicable laws and regulations. The clinical protocol was approved by an independent ethics committee or institutional review board at each participating center. Serious adverse events (AEs) were to be reported to the Drugs Controller General of India (DCGI), the IRB/ethics committee, and the site principal investigator (PI). No vulnerable subject participated in the study. All patients provided written informed consent before study entry.

### 2.2. Patients

Patients were eligible to participate in the trial if they were 18 years of age or older; were diagnosed with IPF within the last 3 years based on the 2002 American Thoracic Society/European Respiratory Society Consensus Statement; had a forced vital capacity (FVC) ≥ 50% predicted; and gave written informed consent. Detailed eligibility criteria are available in Appendix A. Concomitant therapy with nintedanib or pirfenidone was permitted.

### 2.3. Study Protocol

After a screening period, eligible patients were randomly assigned in a 1:1 ratio, with the supplement group receiving Serracor-NK^®^ and Serra Rx260 3 times a day and the placebo group receiving placebo capsules with the same dosing schedule as the supplement group for a period of six months. (The supplements were to be taken on an empty stomach. The dosing schedule is available in Appendix B). Serracor-NK^®^ contains the enteric-coated fibrinolytic enzymes serrapeptase and nattokinase and other proteolytic enzymes, antioxidants, and essential vitamins and minerals, including bromelain, papain, lipase, rutin, amla, coenzyme Q10, and magnesium. Serra Rx260 is comprised of serrapeptase. Patients were randomized using the statistical program in the SAS environment using the random number generation method. The pharmacist dispensed the study treatment (Serracor-NK^®^ + Serra Rx260 or placebo + placebo) per the randomization number. Patients and investigators were unaware of the study-group assignments throughout the study. Three QOL questionnaires: the University of California San Diego’s Shortness of Breath Questionnaire (UCSD-SOB), the Saint George’s Respiratory Questionnaire (SGRQ), and the World Health Organization-5 Well-Being Index (WHO-5), were administered at baseline and at multiple follow-up time points. Physical examination, vital signs, adverse event assessment, concomitant medications, and compliance checks were also conducted at various time points. Laboratory tests were performed at the local labs, and HRCT scans were done as per the standard specifications of each institute at baseline and at the end of treatment. Patients were required to spontaneously report any AEs, including the time of onset and intensity of the event. In addition, AE assessments were performed by the PIs during patient visits. All AEs, independent of the relationship to the study treatment, were to be reported starting from time of first administration until 30 days after the end of treatment, and recorded.

### 2.4. End Points

The primary endpoints were changes from baseline in the total score on the UCSD-SOB, SGRQ, and WHO-5. These self-reported questionnaires measure symptoms, activity, psychosocial impact of the disease, and mental well-being. The secondary endpoint was safety (as assessed by vitals and laboratory tests).

The UCSD-SOB [10] is a questionnaire consisting of 24 items designed to measure self-reported shortness of breath during a variety of activities of daily living (ADL). Each item assesses the severity of SOB on a scale from 0 (none at all) to 5 (maximum or unable to perform due to shortness of breath) across 21 ADL, with three additional questions evaluating fear related to overexertion, limitations, and fear due to SOB. Scores range from 0 to 120, where higher scores indicate more severe dyspnea. A change of 5 units is considered clinically significant for this tool [8]. Hence, a decrease of 5 units or more was considered clinically important improvement, and an increase in score of 5 units or more, clinically significant worsening.

The SGRQ [11] is a standardized self-administered questionnaire specific to airway diseases, comprising 50 items divided into three domains: symptoms (assessing the frequency and severity of respiratory symptoms), activity (measuring the impact of breathlessness on physical mobility and activity), and impact (evaluating the psychosocial consequences of the disease). Scores are weighted, and each domain score and the total score range from 0 to 100, with higher scores indicating greater impairment. A change score of 4 units indicates a slightly effective treatment; 8 units denote moderate efficacy; and 12 units signify high efficacy.

The WHO-5 [12] is a brief, self-reported measure of current mental well-being. It consists of five positively worded statements that individuals rate on a scale from 0 (none of the time) to 5 (all of the time). Raw scores range from 0 to 25, where higher scores indicate better QOL. Percentage scores are calculated by multiplying the raw score by 4. A change in score of 10% is considered clinically significant for this measure.

Vital signs (blood pressure, oxygen saturation, and respiratory rate) were assessed by the clinical staff at each site. Laboratory tests (SGOT, SGPT, and total bilirubin) were assessed at the local lab for each institution. High-resolution CT (HRCT) scans were conducted as per the standard procedures at each enrolling site.

### 2.5. Exploratory End Point

Change from baseline in the extent of fibrosis on HRCT scan. The analysis of HRCT data involved data-driven lung texture analysis (LTA), a machine learning method capable of automatic detection and quantification of lung fibrosis on HRCT. The LTA software performs segmentation and classification on high-resolution CT images. An end-inspiratory scan and a slice thickness of 1 mm or thinner are ideal for the LTA application. The Imbio Lung Texture Analysis (LTA) application (version 2.2.0) was used to visualize and quantify interstitial lung abnormalities and categorize them into radiological reporting categories (ground glass, reticular, and honeycomb). Nine patients in the supplement group and eight patients in the placebo group had both baseline and end-of-treatment scans that met the specifications required to be analyzed by the LTA application and were included in the exploratory endpoint analysis.

### 2.6. Statistical Analysis

Analysis of Demographic Data

Categorical variables are presented as frequencies and percentages calculated according to the number of patients for whom data are available. Continuous variables were summarized using standard measures of central tendency and dispersion, reported as the mean ± SD for normally distributed data. All statistical tests were two-sided and conducted at a significance level of 0.05, with confidence intervals being two-sided at the 90% confidence level. Differences between the treatment and placebo groups were analyzed using the *t*-test for continuous variables and the chi-square test for categorical variables.

For analysis of the primary endpoints, complete data, including baseline and end-of-treatment scores, were available for 48 patients in the supplement group and 40 patients in the placebo group.

## 3. Results

### 3.1. Patients

Between April 2020 and December 2021, a total of 100 patients underwent randomization: 50 in each group. Of these, 48 patients in the supplement group (96%) and 40 patients in the placebo group (80%) completed the study. The most frequent reason for premature discontinuation was worsening of cough, shortness of breath, and fever (1 patient in the supplement group and 6 patients in the placebo group).

The baseline characteristics of patients are summarized in Table 1.

The baseline characteristics of patients in the supplement group and placebo group were similar, with no statistically significant differences in age (62.7 ± 11.9 vs. 60.8 ± 11.8), BMI (23.9 ± 5.1 vs. 25.2 ± 5.3), male:female ratio (22:28 vs. 30:20), use of FDA-approved standard of care medications for IPF (70% vs. 80% of participants), or presence of co-morbidities (28% vs. 26%) between the two groups.

For analysis of the primary endpoints: 48 patients in the supplement group and 40 patients in the placebo group completed the study and had baseline (BL) and end-of-treatment (EOT) scores. Results are presented for these patients:

### 3.2. Primary End Points

#### 3.2.1. UCSD

**Figure 1:**
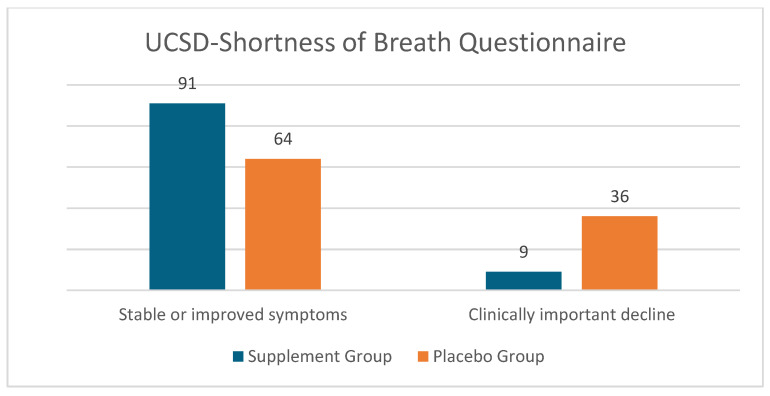
Graphical representation of the effect of Serracor-NK^®^ and Serra Rx260 on University of California San Diego Shortness of Breath (UCSD-SOB) scores in IPF patients that received the supplements vs. placebo (end-of-study scores as compared to baseline).

Of the 48 patients in the treatment group, 13 had no impairment at BL and EOT (a score of 0, indicating no dyspnea) and were not included in the analysis; similarly, of the 40 patients in the placebo group, 15 patients had no impairment at BL and EOT and were not included in the analysis. Thus, the data is presented for 35 patients in the supplement group and 25 patients in the placebo group. As shown in Table 2, there was a statistically significant difference between the treatment vs. placebo group (*p* < 0.05) in the proportion of patients showing clinically important improvement (69% vs. 52%), clinically important decline (9% vs. 36%), and stable symptoms (22% vs. 12%). Figure 1 is a graphical representation of the proportion of patients that had stable or improved symptoms vs a clinically important decline.

#### 3.2.2. SGRQ

**Figure 2:**
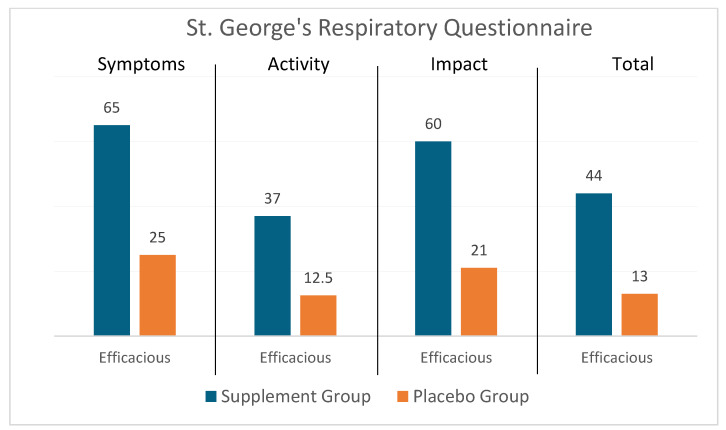
Graphical representation of the impact of Serracor-NK^®^ and Serra Rx260 on St. George’s Respiratory Questionnaire (SGRQ) in IPF patients that received the supplements vs. placebo (end-of-study scores as compared to baseline).

Of the 48 patients in the treatment group and 40 patients in the placebo group, all patients had impairment in the symptom, activity, and total scores at BL or EOT and were included in the analysis. Eight patients in the treatment group and 6 patients in the placebo group had no impairment in the impact score at BL or EOT (a score of 0, indicating no impairment) and were not included in the analysis. As shown in Table 3, there was a statistically significant difference (*p* < 0.01) in the proportion of patients in whom the treatment was very efficacious (46% vs. 20%; 30% vs. 12%; 25% vs. 10%), moderately efficacious (10% vs. 5%; 10% vs. 0%; 2% vs. 0%), slightly efficacious (8% vs. 0%; 20% vs. 9%; 17% vs. 3%), and not efficacious (35% vs. 75%; 40% vs. 79%; 56% vs. 88%) in the symptom, impact, and total domains, respectively, in the treatment vs. the placebo group. The proportion of patients showing improvement in the activity score was not significantly different between the groups (very efficacious: 25% vs. 10%; moderately efficacious: 4% vs. 2.5%; slightly efficacious: 8% vs. 0%; and not efficacious: 63% vs. 87.5%). Figure 2 is a graphical representation of the proportion of patients in whom the treatment was efficacious in the supplement group vs the placebo.

#### 3.2.3. WHO-5

Of the 48 patients in the supplement group, 13 had no impairment at BL and EOT (a score of 25, representing the best possible quality of life) and were not included in the analysis; similarly, of the 40 patients in the placebo group, 13 patients had no impairment at BL and EOT and were not included in the analysis. Thus, the data is presented for 35 patients in the supplement group and 27 patients in the placebo group. As shown in Table 4, the proportion of patients showing clinically significant improvement (60% vs. 56%), clinically significant decline (9% vs. 11%), or no change in mental well-being (31% vs. 33%) as measured by WHO (5) scores was not significantly different between the treatment and placebo groups.

### 3.3. Safety End Points

Vital signs and laboratory parameters are summarized in Table 5. No statistical differences were observed in any of the safety end points between the treatment and placebo groups. Laboratory parameters and vitals were not adversely affected by the treatment intervention and were within the normal range for all participants at baseline as well as end of treatment in both groups.

During the treatment period, 10 patients in the placebo group (20%) and 2 in the supplement group (4%) discontinued treatment due to adverse events. The most frequent reason for premature discontinuation was worsening of cough and shortness of breath, and fever (1 patient in the supplement group and 6 patients in the placebo group). One patient in the supplement group and 3 in the placebo group discontinued due to relocation to a different city, and the remaining one patient in the placebo group found the capsules too big to swallow. The dropout rate was significantly different (*p* < 0.05) between the two groups.

### 3.4. Exploratory Objective

Quantitative analysis of the extent of pulmonary fibrosis: We performed LTA on baseline and end-of-treatment HRCT scans that were analyzable (as per the specifications of slice spacing and thickness of the LTA software) from 9 patients in the supplement group and 8 patients in the placebo group. There was no difference in the percentage of ground glass opacities, reticulation, or honeycombing at baseline or end of treatment between the two groups.

## 4. Discussion

Several novel compounds designed to modify the disease course in IPF are currently undergoing clinical trials, primarily assessing their impact on physiological metrics like lung function and survival. However, the integration of patient-reported outcome measures (PROMs), such as HRQOL scores, has highlighted that the effects of IPF extend beyond respiratory limitations to encompass broader domains of life, including physical, emotional, and social well-being [13]. Therefore, in a relentless disease such as IPF, enhancing HRQOL should be seen as a crucial complement to efforts aimed at enhancing traditional clinical endpoints.

Data synthesized from a systematic review of 134 studies confirmed the quantitative measurability of HRQOL in individuals with IPF [14]. In our study, we used UCSD-SOBQ, SGRQ, and WHO-5 to assess the efficacy of our treatment regimen on HRQOL. The UCSD-SOBQ is validated for monitoring changes in dyspnea over time in IPF patients [3]. The SGRQ is a disease-specific tool assessing the broader impact of airway disease on health, daily activities, and well-being, crucial for quantifying therapeutic effects [15]. The WHO-5, widely accepted for assessing psychological well-being, serves both as a depression screening tool and a clinical trial outcome measure [16].

Despite the availability of disease-specific tools for QOL assessment, research on interventions that enhance HRQOL and functional status in IPF patients remains limited. A review of 34 articles highlighting 19 interventions showed moderate evidence supporting pulmonary rehabilitation and sildenafil in improving QOL [17]. While nintedanib and pirfenidone are effective in slowing lung function decline, they show limited efficacy in alleviating symptoms or improving HRQOL in IPF patients [18,19]. Post hoc analysis suggests pirfenidone may mitigate dyspnea progression in severe IPF cases [20]. To our knowledge, our study represents the first randomized, double-blind placebo-controlled trial investigating the efficacy of systemic enzyme supplementation in enhancing symptoms and QOL in IPF patients.

Over the six-month treatment period, our supplements demonstrated significant efficacy compared to placebo, as evidenced by UCSD-SOB and SGRQ scores. Reductions in UCSD-SOB scores reflect decreased dyspnea severity, which translates to enhanced ability to perform ADLs and engage in social interactions, leading to increased independence and improved QOL. Similarly, decreases in SGRQ scores signify alleviation of respiratory symptoms and enhanced psychological as well as overall well-being, suggesting better management of the impact of IPF on daily life and potentially reduced anxiety and depression associated with chronic illness. Additionally, QOL by SGRQ total is significantly associated with clinical symptoms, number of comorbidities, hospitalization rate, disease severity, and indication for long-term oxygen treatment [21]. Thus, an improvement in SGRQ scores in the supplement group may translate to improved clinical outcomes in these patients. While the duration of our study allowed us to observe significant improvements in symptoms and HRQOL with systemic enzyme supplementation, longer-term studies are necessary to assess sustained benefits and potential long-term effects.

Our selection of Serracor-NK^®^ and Serra Rx260 was guided by historical, anecdotal, preclinical, and clinical evidence highlighting the beneficial effects of their constituent ingredients [7,22,23,24]. Serracor-NK^®^ contains the fibrinolytic enzymes serrapeptase and nattokinase, other proteolytic enzymes, antioxidants, vitamins, and minerals, while Serra Rx260 is comprised of serrapeptase. Serrapeptase is known for its fibrinolytic properties and efficacy in fibrinolytic therapy [25,26], with documented benefits in modulating inflammation [24,27] and enhancing mucus clearance in chronic airway diseases by modifying mucus viscoelasticity and reducing neutrophil count [27]. The fibrinolytic and anti-inflammatory actions of nattokinase are also documented [28]. The observed improvements in dyspnea, cough, and QOL in this study are likely attributable to the fibrinolytic action of these systemic enzymes, facilitating fibrin breakdown in the lungs and aiding mucus clearance.

The supplements Serracor-NK^®^ and Serra Rx260 were well tolerated without apparent interactions with the standard of care drugs—pirfenidone or nintedanib. Vital signs, including oxygen saturation and respiratory rate, were assessed at the various time points as safety measures, along with subjective symptoms and liver function tests. Vital signs are established indicators of clinical status changes [29]. In the present study, LFTs and vitals were not adversely affected by the treatment intervention and were within normal ranges throughout for all participants, indicating the supplements’ safety profile. Previous studies also support the safety of serrapeptase-containing supplements [9,30,31]. Fewer adverse events observed in the supplement group likely contributed to lower dropout rates in this cohort.

IPF is characterized by irreversible accumulation of fibrin in lung interstitial tissue, where an increased extent of fibrotic changes observed on a CT scan serves as a prognostic indicator for mortality [32]. Current treatments are unable to reverse established fibrosis as observed on HRCT scans, which commonly show persistent honeycombing, ground glass opacities, and reticulation [33,34]. Therefore, our exploratory aim was to investigate whether our supplemental regimen could impact the extent of lung fibrosis assessed via lung texture analysis of HRCT scans. Our findings, in the limited number of patients tested, did not reveal differences in the percentage of ground glass opacities, reticulation, or honeycombing between treatment and placebo groups at baseline and end of treatment. The small sample size for HRCT analysis limited the power to detect subtle changes in lung fibrosis parameters. Future studies adopting a standardized HRCT protocol conforming to LTA requirements would facilitate a clearer evaluation of the fibrinolytic activity of these supplements and would provide more robust insights into their potential effects.

## 5. Conclusions

The present study confirms the safety and efficacy of the oral supplements Serracor-NK^®^ and Serra Rx260 in improving symptoms, mental and physical well-being, and functional status, thus enhancing HRQOL in patients with IPF. Our findings highlight the potential of systemic enzyme supplementation to mitigate the multifaceted challenges of IPF. Further evaluations through studies with a larger sample size and a longer duration of 52 weeks or more that can correlate the improvement in HRQOL with physiological function, functional exercise capacity, and extent of lung fibrosis are warranted. Integrating patient-reported outcomes with traditional clinical measures is crucial for a comprehensive evaluation of treatment efficacy and patient-centered benefits in the management of IPF.

## Figures and Tables

**Table 1:** Baseline characteristics of patients.

	Supplement Group (*n* = 50)	Placebo Group (*n* = 50)
Age (years) ± std dev	62.7 ± 11.9	60.8 ± 11.8
Body mass index (kg/m^2^) ± std dev	23.9 ± 5.1	25.2 ± 5.3
Male:Female	22:28	30:20
Patients on SOC IPF medication * (n)	35 (70%)	40 (80%)
Patients with comorbidities (n)	14 (28%)	13 (26%)

* Standard of Care (SOC) IPF medications used were pirfenidone or nintedanib.

**Table 2:** Impact of Serracor-NK^®^ and Serra Rx260 on University of California San Diego Shortness of Breath (UCSD-SOB) scores in IPF patients that received the supplements vs. placebo (end-of-study scores as compared to baseline).

	Supplement Group (*n* = 35)	Placebo Group (*n* = 25)
Participants with clinically important improvement (%)	69	52
Participants with clinically important decline (%)	9	36
Participants with no change (%)	22	12
*p*-Value	0.0205 *	

* significant difference.

**Table 3:** Impact of Serracor-NK^®^ and Serra Rx260 on St. George’s Respiratory Questionnaire (SGRQ): Total, symptoms, activity, and impact scores in IPF patients that received the supplements vs. placebo (end-of-study scores as compared to baseline).

Efficacy	Symptom		Activity		Impact		Total	
	Supplement (*n* = 48)	Placebo (*n* = 40)	Supplement (*n* = 48)	Placebo (*n* = 40)	Supplement (*n* = 40)	Placebo (*n* = 34)	Supplement (*n* = 48)	Placebo (*n* = 40)
Very efficacious (% patients)	46	20	25	10	30	12	25	10
Moderately efficacious (% patients)	10	5	4	2.5	10	0	2	0
Slightly efficacious (% patients)	8	0	8	0	20	9	17	2
Not efficacious (% patients)	35	75	63	87.5	40	79	56	88
*p* Value	0.005 *		0.149		0.005 *		0.008 *	

* significant difference.

**Table 4:** Impact of Serracor-NK^®^ and Serra Rx260 on WHO-(five) well-being index (1998 version) scores in IPF patients that received the supplements vs. placebo (end-of-study scores as compared to baseline).

	Supplement (*n* = 35)	Placebo (*n* = 27)
Participants with clinically significant improvement (%)	60	56
Participants with clinically significant decline (%)	9	11
Participants with no change (%)	31	33
*p*-Value	0.81	

**Table 5:** Vital signs and laboratory parameters in patients with IPF that received the supplements vs. placebo (baseline and end-of-study).

	Supplement Group		Placebo Group	
	Baseline (*n* = 50)	End of Treatment (*n* = 48)	Baseline (*n* = 50)	End of Treatment (*n* = 40)
Mean oxygen saturation	94.4 ± 2.07	96.3 ± 3.08	94.30 ± 2.10	93.60 (3.10)
Respiratory rate	18.40 (2.20)	19.23 (2.14)	18.57 (2.25)	19.83 (2.46)
SGOT	23.84 ± 7.8	25.73 ± 7.7	24.88 ± 10.4	27.1 ± 8.3
SGPT	21.98 ± 8.9	25.7 ± 13	25.4 ± 10.5	29.6 ± 9.8
Total bilirubin	0.5 ± 0.3	0.1 ± 0.3	0.6 ± 0.3	0.7 ± 0.3

## Data Availability

The conditions of our ethics approval do not permit public archiving of the data supporting the conclusions of the study. However, data described in the manuscript and code book will be made available upon request.

## Appendix A

**ELIGIBILITY CRITERIA**

**Inclusion criteria.**

- Clinical diagnosis of Idiopathic pulmonary fibrosis (IPF) within the last 3 years from screening based upon the 2002 American Thoracic Society/European Respiratory Society Consensus Statement.
- Male or female patients aged ≥ 18 years at screening.
- Forced vital capacity (FVC) ≥ 50% of predicted normal.
- Given written informed consent to participate in the study.

**Exclusion criteria.**

Patients with HRCT Pattern of Probable/Indeterminate for UIP.

- Patients currently on Extracorporeal Membrane Oxygenation
- Patients with acute IPF exacerbation or any respiratory tract infection within the four weeks prior to the screening period.
- Alanine transaminase (ALT), Aspartate aminotransferase (AST) > 1.5-fold upper limit of normal (ULN).
- Total bilirubin > 1.5-fold ULN.
- Relevant airways obstruction, i.e., pre-bronchodilator Forced expiratory volume in 1 s/Forced vital capacity < 0.70.
- History of Acute Coronary Syndrome.
- Bleeding Risk:
  ○ Known genetic predisposition to bleeding.
  ○ Patients who require fibrinolysis, full-dose therapeutic anticoagulation or high dose antiplatelet therapy.
  ○ History of haemorrhagic central nervous system (CNS) event within 12 months to screening.
  ○ History of haemoptysis or haematuria, active gastro-intestinal bleeding or ulcers and/or major injury or surgery within 3 months prior to screening.
- Subjects on herbal or mineral preparations for the treatment of IPF.
- Pneumonia diagnosis by High-resolution computed tomography (HRCT) will be performed at the time of screening.
- Planned major surgery during the trial participation, including lung transplantation, major abdominal or major intestinal surgery.
- A disease or condition which in the opinion of the investigator may interfere with testing procedures or put the patient at risk when participating in this trial.
- Alcohol or drug abuse which in the opinion of the treating physician would interfere with the treatment and would affect patient’s ability to participate in this trial.
- Patients not able to understand and follow any study procedures such as but not limited to home spirometry, including completion of self-administered questionnaires without help.
- Women who are pregnant, nursing, who plan to become pregnant while in the trial.
- Women of childbearing potential not willing or able to use highly effective methods of birth control.
- Patients who are or have been participating in another trial with investigational drug/s within one month prior to screening.

## Appendix B

DOSING SCHEDULE:

Directions for Supplement Product TAKE ON AN EMPTY STOMACH (45 min before a meal or 2 h after a meal, with a full glass of water) Step 1. Days 1–4: Take 1 capsule Serracor-NK^®^, 3 times a day (ensure a gap of more than 6 h between supplement intake) Due to the body not being accustomed to systemic enzyme therapy, it is important to start at a minimal dosage and increase as needed. Minor symptoms of intestinal cleansing may occur Step 2. Days 5–8: Take 2 capsules Serracor-NK^®^, 3 times a day (ensure a gap of more than 6 h between supplement intake) At this point, your body should be completed with the cleansing stage and becoming more and more accustomed to systemic enzyme therapy. Step 3. Days 9–176: Take 2 capsules Serracor-NK^®^ + 1 capsule Serra Rx260, 3 times a day (ensure a gap of more than 6 h between supplement intake)
